# Redefining and revisiting cost estimates of routine ART care in Zambia: an analysis of ten clinics

**DOI:** 10.1002/jia2.25431

**Published:** 2020-02-17

**Authors:** Austin Tucker, Tannia Tembo, Radhika P Tampi, Jacob Mutale, Mpande Mukumba‐Mwenechanya, Anjali Sharma, David W Dowdy, Carolyn B Moore, Elvin Geng, Charles B Holmes, Izukanji Sikazwe, Hojoon Sohn

**Affiliations:** ^1^ Department of Epidemiology Johns Hopkins Bloomberg School of Public Health Baltimore MD USA; ^2^ Center for Infectious Disease Research (CIDRZ) Lusaka Zambia; ^3^ University of Alabama Birmingham AL USA; ^4^ Department of Internal Medicine Washington University School of Medicine in St. Louis St. Louis MO USA; ^5^ Center for Dissemination and Implementation Institute for Public Health at Washington University in St. Louis St. Louis MO USA; ^6^ Johns Hopkins University School of Medicine Baltimore MD USA; ^7^ Georgetown University School of Medicine Washington DC USA

**Keywords:** AIDS, HIV, antiretroviral therapy, costs and cost analysis, low‐resource setting, Zambia

## Abstract

**Introduction:**

Accurate costing is key for programme planning and policy implementation. Since 2011, there have been major changes in eligibility criteria and treatment regimens with price reductions in ART drugs, programmatic changes resulting in clinical task‐shifting and decentralization of ART delivery to peripheral health centres making existing evidence on ART care costs in Zambia out‐of‐date. As decision makers consider further changes in ART service delivery, it is important to understand the current drivers of costs for ART care. This study provides updates on costs of ART services for HIV‐positive patients in Zambia.

**Methods:**

We evaluated costs, assessed from the health systems perspective and expressed in 2016 USD, based on an activity‐based costing framework using both top‐down and bottom‐up methods with an assessment of process and capacity. We collected primary site‐level costs and resource utilization data from government documents, patient chart reviews and time‐and‐motion studies conducted in 10 purposively selected ART clinics.

**Results:**

The cost of providing ART varied considerably among the ten clinics. The average per‐patient annual cost of ART service was $116.69 (range: $59.38 to $145.62) using a bottom‐up method and $130.32 (range: $94.02 to $162.64) using a top‐down method. ART drug costs were the main cost driver (67% to 7% of all costs) and are highly sensitive to the types of patient included in the analysis (long‐term vs. all ART patients, including those recently initiated) and the data sources used (facility vs. patient level). Missing capacity costs made up 57% of the total difference between the top‐down and bottom‐up estimates. Variability in cost across the ten clinics was associated with operational characteristics.

**Conclusions:**

Real‐world costs of current routine ART services in Zambia are considerably lower than previously reported estimates and sensitive to operational factors and methods used. We recommend collection and monitoring of resource use and capacity data to periodically update cost estimates.

## 1 Introduction

In the last decade, Zambia has restructured much of the way it provides ART care in the form of task‐shifting by increasing the role of nurses (e.g. initiating treatment and prescribing medications) and CHWs (e.g. taking vitals and restocking/dispensing drugs), thereby enabling decentralization of treatment initiation and management of ART to peripheral health centres and changes in treatment guidelines 1, 2, 3. Additionally, with developments of new regimens and increased shifting to generics as well as strong donor support, ART drug prices have greatly decreased over the last decade 4, 5. With the adoption of the World Health Organization’s (WHO) expanded ART eligibility, the percentage of people receiving ART has increased sharply in high burden countries; for example, Zambia has reported a 25% increase since 2011 6. These major changes in health service delivery have direct impact on costs.

Costs of public health programmes have been an integral component of informed decision‐making at various donor agencies, including the Impact and Efficiency Acceleration Plan of the President’s Emergency Plan for AIDS Relief (PEPFAR) 7. Furthermore, with the ambitious WHO 90‐90‐90 targets by 2020 and 95‐95‐95 targets by 2030 8, 9, understanding the key drivers (e.g. operational factors, resource components and clinical characteristics) of the costs of routine and alternative strategies such as differentiated service delivery (DSD – strategies for providing various ART services throughout the care cascade at the community level or during facility off‐hours to increase access and reduce typical burdens of engaging with ART care) 10, 11, 12, 13, 14 is a key part of the assessment of programme feasibility, cost‐effectiveness and sustainability.

As a first step in health economic evaluation of ART provision, it is critical to have an up‐to‐date understanding of costs of routine ART care. However, with vastly revised ART guidelines and changes in drug prices, existing evidence on costs are out of date 15, 16, 17. Moreover methodologies employed by earlier studies are variable and not fully transparent 18. As Zambia considers expanding to universal testing and treatment, ART coverage will expand, further supporting the importance of understanding the costs of providing ART treatment 19.

To address these limitations and update current ART service costs in Zambia, we collected and compared an extensive set of primary cost and operational data at ten public ART clinics in evaluating current routine ART service costs. Furthermore, we explored different costing methods and analytic perspectives (facility‐level vs. individual patient) to identify attributes contributing to differences in cost estimates. Subsequently, our study aims to increase transparency and standardization of costing methodologies employed in the future studies.

## 2 Methods

We assessed costs using both bottom‐up and top‐down methods to identify key contributing factors in the cost differences in the two distinct methods. Ultimately, we aimed to report the real‐world cost estimates for current ART service costs that can be utilized as important base‐case estimates for cost‐effectiveness analyses of interventions aiming to improve ART service delivery. This study was part of a parent study, the Community ART for Retention (CommART) programme, which examined the effectiveness of differentiated service delivery models for ART care in Zambia.

### 2.1 Setting and site selection

We used purposive sampling to select 10 of the 26 clinics engaged in the CommART study to reflect representation across four key clinic characteristics: ART patient volume (based on the total number of clinic visits), geographical representation (based on a binary rural/urban distinction), clinic population (based on the unique patient volume) and retention (based on the cumulative incidence of missed visits ≥14 days late).

### 2.2 Measurements

#### 2.2.1 Time and motion data

Our study utilized an activity‐based costing framework using time‐and‐motion (TAM) data measuring observed direct and indirect (defined based on whether or not healthcare worker activities involve direct interaction with patients and/or clinical specimens) service times of key healthcare workers (HCWs) – counsellors, nurses, clinical officers and pharmacy technicians – as the primary cost allocation criteria 20. As TAM studies capture operational variabilities within and across the clinic staff and ART clinics by types of activities through direct observation, use of TAM data in cost analysis allows for more direct empiric assessment of activity‐based costs compared to alternative tools such as periodic effort survey or self‐reported time sheets.

#### 2.2.2 Primary cost data

Primary cost and operational data – assessed as aggregated annual cost over two full fiscal years between 2015 and 2016 – from the ten clinics were collected using a standardized health facility cost data collection tool. This tool included resource‐use data on clinic operations, staffing, physical space, and administrative and district‐wide supervision. Maintenance, building space and capital assets were annuitized based on their respective useful life years. Details on the data collected for each resource categories are available in the appendix.

As we obtained the annual overhead costs for the facility where the ART clinic occupied space, we apportioned the overhead cost dedicated to ART care based on the size and volume of ART patients seen at the clinic relative to other services offered at the facility (expressed as %ART of Total Services, shown in Table 1).

**Table 1:** Clinic characteristics

Clinic^a^		Clinic 1	Clinic 2	Clinic 3	Clinic 4	Clinic 5	Clinic 6	Clinic 7		Clinic 8	Clinic 9		Clinic 10
Urban versus rural		Urban	Urban	Urban	Urban	Urban	Urban	Rural		Rural	Rural		Rural
Facility integration		Not integrated	Not integrated	Service and space integrated	Service and space integrated	Not integrated	Not integrated	Service and space integrated		Service and space integrated	Service and space integrated		Service and space integrated
Staffing^b^													
Clinical													
Counsellors		3	7	4	1	3	7	5		4	4.5		1
Clinical officers		2	2	4	5	1	2	–		1	0.5		–
Nurses		6	1	7	10	3	1	1.2		1	1		4.9
Pharmacy technician		3	2	2	3	3	2	1		2	0.4		1
Laboratory technician		0	0	2	0	1	2	4.5		0	0.5		1.2
Non‐clinical		7	4	1	1	8	8	1		1	0		0.9
Total		21	16	20	20	19	22	12.7		9	6.9		9
Service statistics													
Unique patient volume 2016		9104	8050	6410	5127	4597	3094	3080		1076	873		477
Total clinic visits 2016		50830	35148	28354	16857	20988	12571	13067		4976	3043		1947
Average annual visits per patient		5.58	4.37	4.42	3.29	4.57	4.06	4.24		4.62	3.49		4.08
Average clinical visits per patient		2.24	3.07	2.56	2.74	2.70	1.93	1.57		2.31	1.50		1.32
Average pharmacy visits per patient		5.24	4.00	3.81	1.28	4.15	3.79	2.80		4.33	2.68		3.45
% ART of total services		35%	49%	54%	32%	29%	28%	39%	43%			35%	7%
Daily workload/staffing ratios^b^													
Daily patient/counsellor ratio		65	19	27	65	27	7	10	5			3	7
Daily patient/clinical officer ratio		97	67	27	13	80	24	N/A	19			23	N/A
Daily patient/nurse ratio		32	135	16	6	27	48	42	19			12	2
Daily patient/laboratory technician ratio		N/A	N/A	54	N/A	80	24	11	N/A			23	6
Daily patient/pharmacy technician ratio		65	67	54	22	27	24	50	10			29	7

Clinic characteristics for the clinics as collected through various tools and described in greater detail in the appendix (under “Clinic Characteristics”).

a We did not include clinic names; however, clinic‐specific data, including the names, are available on request to the authors

b staffing calculations are done measuring the number of full‐time employees dedicated to ART care. If clinic staff indicated they work only some percentage of their time in the ART clinic, then they were allocated as the equivalent fraction of an employee. Daily ratios were calculated as the average number of patients visiting the facility for ART care, divided by the number of full time equivalent employed staff at the facility. This may not equate to the number of patients seen by one staff member on an average day, as not all patients will interact with staff (of a given type) at each visit.

To collect data on unit prices of key medical consumables, drugs and supplies, we extracted prices from a catalogue compiled by the Medical Stores Limited (MSL) in Zambia 21. In Zambia, ART drugs are procured through the Ministry of Health (MoH) and a Procurement and Supply Management (PSM) team consisting of donors, implementing partners, the Medical Stores Limited (MSL) and MoH pharmacists. ART drugs are then received and stored by the Medical Stores Limited who distributes the drugs to hospitals and districts based on their orders. These prices are quoted in the catalogue as the average of the cost for procuring drugs for the PSM team. These prices were compared with those reported in the WHO Global Price Reporting Mechanism to ensure that our ART prices were similar to others being reported for 2016. Unit prices were then computed based on the smallest service delivery unit (e.g. individual dose/pill of ART medication) used in our cost analysis.

#### 2.2.3 Service utilization data

To evaluate annual drug and ART service costs, we queried service utilization and drug dispensing data from SmartCare, an electronic health record system (EHR) used to provide continuity of care in Zambian government health facilities. In ART clinics, SmartCare contains patient‐level information on dates of patient enrolment and ART initiation, drugs dispensed, types of clinic visits, scheduled clinic appointments and results of laboratory tests. These data were used to compile baseline costing metrics for the number of visits per year. Visits were categorized as clinical and non‐clinical visits, based on a documented encounter with a clinical officer (or equivalent). We assumed that all patient visits to the clinic included counselling and pharmacy visits. Overall facility‐level data were validated by comparison to patient‐level data on the annual average number of clinic visits, drug dispensation and laboratory tests queried from the SmartCare database for a random sample of 100 patients prescribed ART in each facility (1000 ART patients total).

### 2.3 Cost analysis

All costs were converted to 2016 US dollars (USD); pre‐2016 cost data were adjusted to year 2016 currency based on the GDP deflator for Zambia and converted using the 2016 Zambian kwacha‐USD currency exchange rate 22, 23.

#### 2.3.1 Bottom‐up costs

For generalizability and simplicity, all costs were categorized into six key elements of resources, including: staff, building, overheads, supplies, drugs and laboratory costs. These resource categories were reported for each of the seven clinical activities observed in the ART clinic from our time‐and‐motion data, including: clinic, counselling, pharmacy and laboratory visits, administrative, triage and other activities.

#### 2.3.2 Annual costs

All costs were first assessed as annual costs and used to calculate costs‐per‐minute based on the total annual operational time for each clinic. These cost‐per‐minute figures (for staff time and room/capital asset time) were then multiplied by data from the time‐and‐motion study to produce a distribution of costs‐per‐activity depending on the time spent performing the seven clinical activities. Indirect patient activity costs (administrative and other activities) were calculated using the same method and then re‐distributed into each direct patient activity (clinic, counselling, pharmacy and laboratory visits and triage) costs based on the percentage of overall time spent by clinic staff. Drug costs were assessed as part of the pharmacy visit costs, while supplies were included as part of the clinic visit costs.

We first calculated each activity cost as a cost‐per‐visit, which was stratified by resource type. To extrapolate annual service costs, activity/resource type costs were then multiplied by the corresponding mean annual number of visits per patient at each clinic. Capital assets were annuitized over their expected useful lifetime with a 3% discount rate 24.

#### 2.3.3 Drug costs

Total annual cost of ART drugs at each clinic was assessed based on unit prices multiplied by the total number of pills dispensed at the clinic. This cost was then divided by the total number of unique patient clinic visits to compute an average annual ART drug cost per patient. Costs for supplies and laboratory were similarly assessed using an ingredients approach.

We applied different assessment criteria in evaluating the drug costs using both the facility and patient level data. The facility‐level data featured the total amount of ART medication dispensed by each clinic, while the patient‐level data represented a random sample of 100 ART patients from each clinic – including information about the number of ART medications received.

We compared overall drug costs to previous studies that have investigated ART costs in Zambia. While most previous studies examined drug costs on a per‐patient basis, we primarily looked at drugs as they were dispensed at the facility‐level. We then compared the difference in both utilization of drugs and the price of drugs to understand how much differences in overall costs of ART were due to price changes or utilization changes.

#### 2.3.4 Top‐down costs

Top‐down costs were calculated using the same aggregate annual cost data for overhead, capital assets and staffing at each of the clinics. This figure for each clinic was divided by the number of unique ART patients who visited the ART clinic during the 2016 calendar year to calculate the annual ART services cost per patient. Costs of drugs, supply or laboratory were added to this estimate based on the method described above.

#### 2.3.5 Missing capacity costs

We anticipated that differences in unit cost estimates using top‐down and bottom‐up methods would be sensitive to how costs associated with operational inefficiencies and non‐patient activities are captured. We first calculated the daily cost of clinic operations (overhead + staffing + building costs) from our total annual clinic operating cost estimate. Then, we assessed the trend of daily patient service volumes (PSV) at each clinic and established an average capacity based on the distribution of PSV throughout the year. We anticipated that most staff time would be devoted to direct patient care on high‐volume days, and to administrative tasks or other activities such as evaluating paediatric ART patients on low volume days. Therefore, we assessed the gap between each clinic’s capacity for high PSV and low PSV days as the cost associated with under‐utilized capacity, considering a range of different capacity thresholds for sensitivity analysis (see Appendix Table S3). In our primary analysis, we used the 95th percentile of the PSV for each clinic as the threshold for a high capacity day. An operational cost was allocated per patient seen less than that threshold. This per‐day cost was summed across one year to provide an annual “missing capacity” cost for each clinic. For comparative purposes, the total annual missing capacity cost was divided by the number of unique ART patients to assess the contribution of this cost in the gaps in the annual cost per patient on ART assessed using two different costing methods.

### 2.4 Sensitivity analysis

Although our clinic sample size was not powered to produce statistically meaningful estimates, we performed an exploratory analysis using ordinary least‐squares regression to examine the relationships between operational factors and ART costs. We used univariate regression in which individual clinic characteristics (listed in Table 1) were the independent variables and average annual cost per patient was the dependent variable.

## 3 Results

### 3.1 Site selection

Clinic volume ranged from 1947 to 50830 annual clinic visits, including 477 to 9104 unique ART patients. Average full‐time employees (including fractions of full‐time employees who reported spending only some of their time in the ART clinic) varied widely between clinics, from 6.9 to 22. Additionally, the mean daily patient‐to‐staff ratio varied according to staff role (Ranges: Counsellors: 3 to 65, Clinical Officers: 13 to 97, Nurses: 2 to 135, Laboratory Technicians: 6 to 80, Pharmacy Technicians: 7 to 67), suggesting differential composition of full‐time ART staff in each clinic. A full summary of clinic characteristics is presented in Table 1.

### 3.2 Per patient costs

#### 3.2.1 Bottom‐up cost analyses

**Figure 1:**
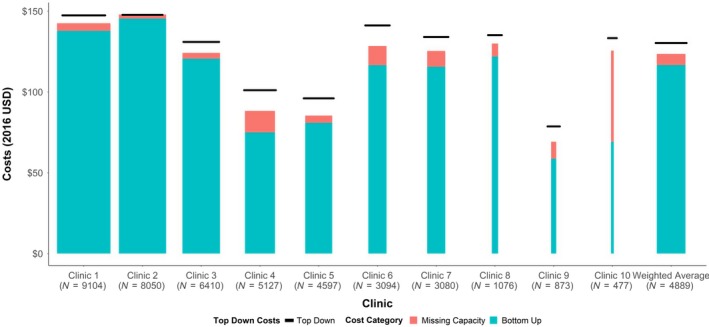
Average annual cost per patient in ART care by clinic, including missing capacity costs and top‐down costs. Green bars represent the estimated annual per‐patient ART cost, using a bottom‐up costing methodology. The red column represents the missing capacity costs as determined by a 95% capacity threshold, and the small horizontal black lines represent the corresponding estimates of ART costs per patient year, using a top‐down cost estimate Missing capacity were calculated by examining daily patient volumes of the clinics and allocating additional operational costs of the days when the clinic is seeing below its maximum capacity, calculated as the 95th percentile of the daily patient volume distribution for each clinic. The width of each bar (except the “weighted average” bar) represents the relative volume of patients on ART in each clinic; the weighted average is weighted by the unique patient volume for each clinic (i.e. the total number of unique patients who visited the ART clinic in one 2016 depicted under the clinic name).

Combining the TAM data with our time‐associated cost summaries, we estimated that the annual per‐patient ART service costs varied at the clinic level from $58.75 to $145.44, with an overall mean of $116.69 per patient per year (Figure 1, Table 2). The weighted average number of total annual visits per patient was 4.48 visits (range: 3.29 to 5.58) with an average cost per visit of $26.02 (range: $16.85 to $33.31). At all sites, ART drug cost was the largest cost component with an average per‐patient annual cost of $88.24 (range: $40.02 to $127.23), followed by laboratory costs of $17.00 (range: $1.88 to $30.09), supply costs of $6.05 (range: $1.28 to $16.94), and direct staff costs of $4.15 (range: $2.76 to $8.20). When comparing these estimates by geography, the annual per‐patient cost was $14.81 (range: ‐$46.97 to $79.11) higher in urban than in rural clinics ($118.64 vs. $103.83). This difference was mostly attributable to differences in cost associated with laboratory investigations ($15.31 vs. $6.46).

**Table 2:** Annual mean per‐patient ART costs (in 2016 USD) by clinic, bottom‐up costing

	Clinic 1	Clinic 2	Clinic 3	Clinic 4	Clinic 5	Clinic 6	Clinic 7	Clinic 8	Clinic 9	Clinic 10	Weighted average
Staff	$3.85	$4.83	$4.21	$2.80	$4.95	$3.82	$3.74	$8.20	$2.76	$2.76	$4.15
Building	$0.54	$0.51	$0.28	$0.26	$0.35	$0.37	$2.37	$3.26	$0.15	$0.14	$0.62
Overhead	$0.18	$0.82	$0.53	$0.32	$0.46	$0.36	$2.11	$1.73	$1.18	$0.69	$0.63
Supplies	$16.94	$0.52	$7.17	$1.64	$1.28	$3.70	$1.10	$8.74	$9.73	$4.33	$6.05
Drugs	$86.26	$127.23	$92.36	$55.40	$63.87	$80.45	$97.62	$96.70	$40.02	$59.39	$88.24
Laboratory	$30.09	$11.53	$16.06	$14.57	$10.00	$27.91	$8.70	$3.33	$4.91	$1.88	$17.00
Total	$137.86	$145.44	$120.61	$74.99	$80.91	$116.61	$115.64	$121.96	$58.75	$69.19	$116.69
Per visit	$24.69	$33.31	$27.27	$22.81	$17.72	$28.70	$27.26	$26.37	$16.85	$16.95	$26.02

#### 3.2.2 Top‐down versus bottom‐up cost estimates

Compared to bottom‐up estimates, annual per‐patient ART service costs were consistently higher when using top‐down methods (Figure 1). Variability in top‐down ART service costs (range: $69.06) was smaller than the bottom‐up estimates (range: $86.69). Urban and rural clinics had similar estimated annual per‐patient costs by the top‐down method ($131.06 vs. $125.44). Overall, we found that on average the missing capacity costs represented 55% (range: 30% to 100%) of the difference between the top‐down and bottom‐up cost estimates (Figure 1).

### 3.3 Drug costs

Annual ART drug costs per patient across the ten clinics included in our study varied from $40.02 to $127.23 per patient (Table 3). When using patient‐level data (a random sample of 100 active ART patients in each clinic) we found that the annual ART drug costs were generally higher, ranging from $61.31 to $122.02 per patient. Long‐term patients were found to have higher costs ($64.82 to $133.33 per patient) than “recently initiated” patients who had been diagnosed and initiated on ART in the previous year ($29.74 to $133.25).

**Table 3:** Annual per‐patient ART drug costs by clinic

Clinic	Facility level data^a^	Patient level data		
		Overall	Long‐term ART patients	Recent initiation^b^
Clinic 1	$86.26	$105.60	$116.34	$44.77
Clinic 2	$127.23	$95.86	$107.45	$61.09
Clinic 3	$92.36	$89.71	$96.42	$48.50
Clinic 4	$55.40	$70.24	$70.98	$65.28
Clinic 5	$63.87	$117.55	$133.33	$67.60
Clinic 6	$80.45	$99.27	$108.45	$54.72
Clinic 7	$97.62	$109.39	$114.56	$77.62
Clinic 8	$96.70	$122.02	$121.18	$133.25
Clinic 9	$40.02	$61.31	$64.82	$29.74
Clinic 10	$59.39	$72.39	$77.64	$51.40
Weighted Average	$88.24	$95.40	$105.79	$58.68

a Facility level data is the based on the complete number of drugs dispensed by each clinic

b Recent initiation patients are patients retained in ART care for <1 year of time, long‐term patients are anyone retained in care longer than 1 year.

### 3.4 Regression

Within this sample of ten clinics, the annual per‐patient cost was significantly associated (in univariate analysis) with the total number of clinic visits: total per‐patient costs increased by $1.30 (95% CI: $0.10 to $2.49) for each additional 1000 visits (Table 4). Other factors associated with annual per‐patient costs included the volume of unique ART patients ($6.39 per 1000 patients; 95% CI: −$0.01 to $12.70), the average number of visits per patient ($32.59 per additional visit; 95% CI: $3.64 to $61.54), the daily patient: nurse ratio ($0.52 per each unit increase in patient:nurse ratio, 95% CI: $0.03 to $1.01) and the daily patient: pharmacy‐technician ratio ($0.94 per unit increase in patient:pharmacy technician ratio, 95% CI: $0.10 to $1.78). Additional regression results are given in Table 4.

**Table 4:** Increase in annual per‐patient cost of antiretroviral therapy (ART) with one‐unit changes in key selected parameters

Variable	Change in annual cost of ART services per patient (95% confidence interval)	*p*‐Value
Geography (1 = Urban 0 = Rural)	$21.35 (−23.60 to 66.30)	0.31
Total number clinical visits (per 1000 patients)	$1.30 (0.10 to 2.49)	0.04
Unique patient volume (per 1000 patients)	$6.39 (−0.01 to 12.7)	0.05
Percent of clinical services dedicated to ART	$52.67 (−6.07 to 299.1)	0.06
Average number of total annual visits per patient	$32.59 (3.64 to 61.54)	0.03
Average number of annual clinical visits per patient	$21.96 (−15.93 to 59.85)	0.22
Average number of annual pharmacy visits per patient	$17.24 (−0.84 to 35.32)	0.06
Patients/counsellor ratio (per 1 additional patient per staff role)	$0.16 (−0.89 to 1.21)	0.73
Patients/clinical officer ratio (per 1 additional patient per staff role)	$0.38 (−0.52 to 1.27)	0.34
Patients/nurses ratio (per 1 additional patient per staff role)	$0.52 (0.03 to 1.01)	0.04
Patients/pharmacy technician ratio (per 1 additional patient per staff role)	$0.94 (0.10 to 1.78)	0.03
Patients/laboratory technician ratio (per 1 additional patient per staff role)	$0.07 (−1.26 to 1.40)	0.89
Clinical full time employees	$2.32 (−3.47 to 8.10)	0.38
Total clinic full time employees	$2.12 (−1.90 to 6.14)	0.26

## 4 Discussion

Ascertainment of current costs and factors influencing ART service delivery from the perspective of a functioning ART clinic serves at least two important priorities 10, 18. First, it provides an evidence base for future expenditure requirements that can be projected based on current resource mobilization and expected growth in operations. Second, it can serve to identify areas for improvement when designing strategies to improve the cost‐efficiency of ART service delivery.

Using a wide range of data sources, costing methods and comparative review of existing literature, we report that the current cost of routine ART service delivery in Zambia is considerably lower than that reported in 2011 and earlier 16, 17, 25. We factored the changes in the global ART drug price reduction in the past decade using the WHO Global Price Reporting Mechanism and monthly drug prices used in the published studies in Zambia 24, 26. We found that the effect of drug price reductions on the overall ART services costs is important (see appendix), but the price reduction only explains 50% of the difference in our estimated per‐patient‐year drug costs ($88.24, range: $40.02 to $127.23 and a previous estimate ($159, range: $116 to $207 per patient year) from 2011 16. The remainder of this difference was likely attributable to changes in drug utilization, including reductions in per‐patient utilization of ART drugs, recommended regimen changes and the costing methodology employed. We do not have data on the cost of distribution of drugs from the Medical Stores Limited to individual facilities. These costs may be included within the price listed in the catalogue; however, because we did not include any mark up, these drug costs may be an underestimation. Changes within the drug procurement system and amongst the composition of the PSM team may also be a contributing factor to reductions in the price of ART drugs within Zambia.

Cost estimates are sensitive both to methods and data considered in the analysis. We first demonstrate this by comparing our top‐down and bottom‐up cost estimates, illustrating that the difference in these estimates is mostly explained by how one accounts for costs associated with operational inefficiencies (missing capacity costs). Furthermore, using multiple analytic criteria in calculating annual per‐patient ART drug costs, we show that the costs are highly variable to inclusion and exclusion of types of ART patients (long‐term vs. newly initiated ART patients) and data sources (facility vs. patient‐level). This observed variability should be carefully assessed when planning and projecting the economic impact of ART and similar public health interventions.

As operational factors are strong determinants of per‐patient ART costs 18, we further investigated how ART service costs are influenced by key operational factors observed at the study sites. In our regression analysis, we find that proximal determinants (patient volume, patient to staff ratios) partially explain differences in the costs observed across the ten clinics, but the main source of between‐clinic variability represents variation in drug costs. Our findings are consistent with findings reported by Menzies and colleagues in 2012 with patient volume being strongly associated with lower per‐patient costs. While marginally significant, our analysis also indicates that increases in workload – as measured by lower health staff to patient ratio – for both nurses and pharmacy technologists are associated with lower costs. These results may reflect economies of scale at play in the health facilities; however, we lack the longitudinal data or sample size to perform the necessary analysis to causally estimate the effects economies of scale on costs 27. Likewise, it is important to note that per‐patient cost estimates do not accurately reflect issues in workload constraints nor the quality of care. Therefore, relevant decision makers and analysts should comparatively and comprehensively evaluate patient outcomes, programme quality, sustainability and coverage when assessing such issues as the ideal staff‐to‐patient ratio.

While we rigorously assessed a wide range of data and employed multiple costing methods to address uncertainties, several limitations should be noted. First, our focus was on patients receiving ART services; as such, we did not examine the costs for patients who are not receiving ART. Our findings, therefore, may not generalize to those patients. Second, while facilities were purposively sampled to be representative of the overall set of facilities in the trial, we cannot be certain that these findings would generalize to all clinical facilities across Zambia. Uncertainties in our estimates are primarily assessed as the aggregate of costs and operational factors in the ten clinics included in this study, so, we are not able to assess within‐clinic cost variability resulting from day‐to‐day differences in clinic operations. There are existing heterogeneities across the facility operations in terms of clinic integration which may affect the unit costs via economies of scope due to integration in space and services between OPD and ART services. We are unable to causally estimate the effect of economies of scope on the unit cost of ART service delivery, but this may influence the generalizability of the results to other settings. Additionally, our regression analysis should be considered exploratory, and the generalizability of our cost model in predicting per‐patient ART costs in other settings taken with a measure of caution, given our small sample size of ten clinics and limited number of variables tested in our model. Ultimately, our assessment of factors contributing to ART service costs utilized a cross‐section of select clinics; therefore, it is difficult to quantify the relative causal contributions of factors that are associated with variability in costs and/or the overall reduction of ART costs in Zambia.

## 5 Conclusions

Our study provides an empirical basis for evaluating costs of ART care in resource‐limited settings and demonstrates the importance of using a generalizable and transparent cost analysis structure to allow assessment of the determinants of ART service costs. With the rapidly changing operational and global market landscape for HIV/AIDS care, service delivery costs should be monitored consistently in a transparent manner. This, in turn, will allow for efficient planning and accurate assessment of the health economic impact of improved and innovative service delivery models that may be critical to achieving global targets for HIV/AIDS control.

## Competing Interests

There are no existing competing interests involved in this study.

## Authors' contributions

AS, MM, DD, CM and HS designed the research study. AT, TT, RT, JM and HS collected the data. HS, RT and AT performed analysis of the data and designed essential tools. AT and HS wrote the paper. EG, DD, IS, CH, and CM provided feedback and oversight of the research, the paper and the overarching trial.

## Abbreviations

AIDS, acquired immunodeficiency syndrome; ART, antiretroviral therapy; EHR, electronic health record; HCW, health care worker; HIV, human immunodeficiency virus; MoH, Ministry of Health; MSL, Medical Stores Limited; PSM, Procurement and Supply Management; PSV, patient service volume; TAM, time‐and‐motion; USD, United States Dollar; WHO, World Health Organization.

## Supporting information


**Figure S1.** ART dispensary NRTI:NNRTI ratios by clinic

**Figure S2. **Annual bottom up cost per patient by cost category

**Appendix S1.** Supplementary materials
**Table S1.** Facility characteristics
**Table S2.** Missing capacity costs by missing capacity threshold
**Table S3.** Clinic costs by geography
**Table S4. **Univariate regression outputs on dependent variable: annual operational cost
