# Supplementary figures and images for: Redefining and revisiting cost estimates of routine ART care in Zambia: an analysis of ten clinics
Source: J Int AIDS Soc. 2020 Feb 17;23(2):e25431. doi: 10.1002/jia2.25431 (PMC7025092; doi:10.1002/jia2.25431)

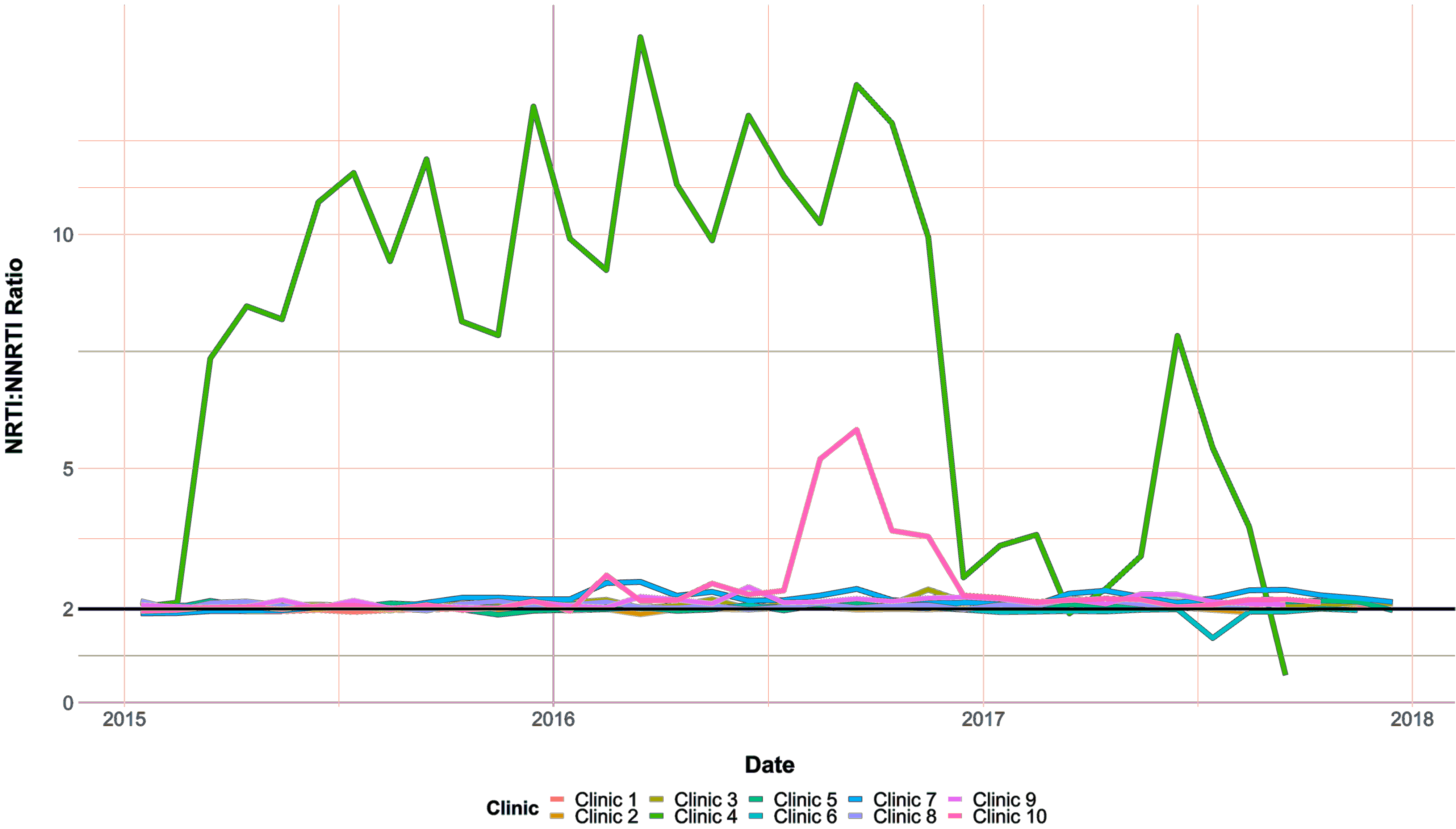

Supplement: Supplementary file 1 — Figure S1. ART dispensary NRTI:NNRTI ratios by clinic [file JIA2-23-e25431-s001.pdf]

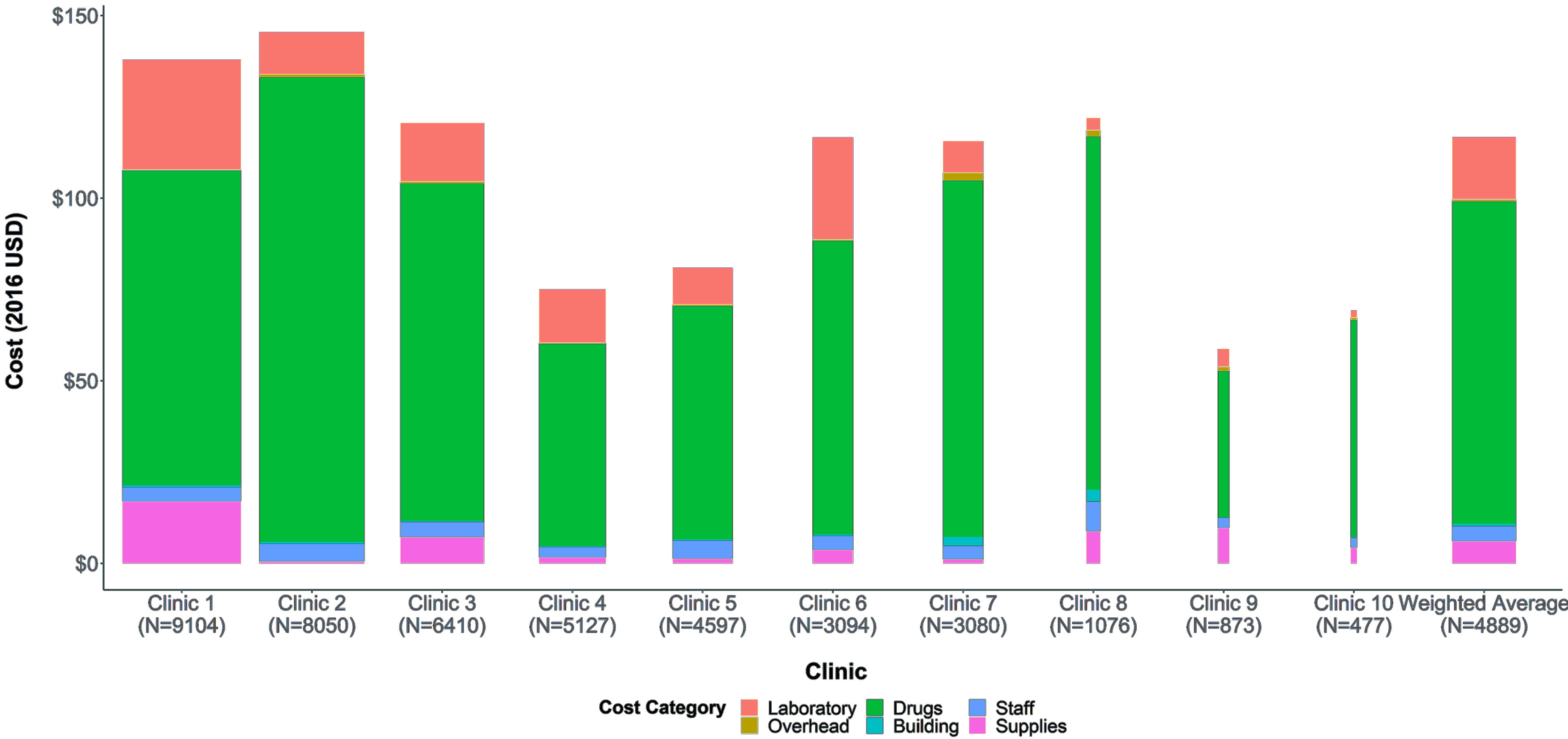

Supplement: Supplementary file 2 — Figure S2. Annual bottom up cost per patient by cost category [file JIA2-23-e25431-s002.pdf]
