# Supplementary material for: Redefining and revisiting cost estimates of routine ART care in Zambia: an analysis of ten clinics
Source: J Int AIDS Soc. 2020 Feb 17;23(2):e25431. doi: 10.1002/jia2.25431 (PMC7025092; doi:10.1002/jia2.25431)
Supplement: Supplementary file 3 — Appendix S1. Supplementary materials Table S1. Facility characteristics Table S2. Missing capacity costs by missing capacity threshold Table S3. Clinic costs by geography Table S4. Univariate regression outputs on dependent variable: annual operational cost [file JIA2-23-e25431-s003.docx]

**Appendix:**

The purpose of this appendix is to provide supplementary information on the methodology and analyses performed.

**Facility Selection**

To have an even distribution of facilities in this sub-group analysis, selection of facilities was based on total clinic population, geographic location (urban vs rural), and adherence as measured by the cumulative incidence of a missed visit (> 14 days late). We aimed to have a representational selection of clinics across the different intervention arms from the larger CommART study evaluating different DSD models. **Table S1**, contained below, depicts the breakdown across the selected clinics.

**Table S1: Facility Characteristics**

| Name of the study clinic | Intervention/Control | Urban/Rural | Clinic Population | cumulative incidence of missed visit  (> 14dy late) |
| --- | --- | --- | --- | --- |
| Community Adherence Group (CAG) | | | | |
| Kalomo District Hospital | Intervention | Rural | 2890 | 0.36 |
| Nsadzu | Intervention | Rural | 699 | 0.47 |
| Mwase Lundazi | Control | Rural | 545 | 0.48 |
| Mbayamusuma | Control | Rural | 1014 | 0.32 |
| Urban Adherence Group (UAG) | | | | |
| Kabwata | Intervention | Urban | 3757 | 0.50 |
| Kalingalinga | Intervention | Urban | 7502 | 0.40 |
| Nangongwe | Control | Urban | 3067 | 0.48 |
| Chelstone | Control | Urban | 8402 | 0.43 |
| Fast Track (FT) | | | | |
| Makeni | Intervention | Urban | 4720 | 0.35 |
| Matero Main | Intervention | Urban | 5048 | 0.46 |

Facility characteristics showing treatment assignment, urban/rural designation, clinic population, and adherence measured as cumulative incidence of missed visits. These were the main criteria used for facility sample selection for the baseline cost data collection.

**Clinic Characteristics**

Unique patient volume is the unique number of patients that went to the clinic for ART care in 2016. The total clinical visits per patient are the total number of visits in which a patient had an interaction with a clinician, received ART counseling, and collected drugs from the pharmacy. Pharmacy visits were defined as a patient receiving both pharmacy and counseling services. The percent ART services (%ART Services) figure was calculated by dividing the total ART patient volume by a combined total volume for the entire clinic including in-patient days, surgeries, deliveries, ART services, etc. This measurement serves as a tool for distributing the total overhead costs of the clinic to just ART services. The total staff at each facility were calculated by summing the total number of each type of role through our overhead costing tool. If staff specified they only allocated some proportion of their professional time at the ART clinic, then they were included as only a proportional fraction of an employee (e.g. if a staff member reported spending only 50% on ART services, then they were counted as 0.5 an employee). Daily workload ratios are calculated by dividing the total number of patient visits by the number of operational days and dividing that by the number of each type of staff. N/A means that there were no staff of that type listed at that clinic, and thus the ratio is undefined.

**Staff Costs**

Staff costs were primarily calculated using our time-and-motion data as well as staff salaries. Salaries were estimated based on government salary grades which were collected by a CIDRZ research staff member. All salary grades were provided as a range, so for the primary analysis, the salary values used were the average between the low and high values for possible salary. Additionally, the same position may have been on different salary grades across different facilities or even at the same facility (e.g. pharmacy technicians at one facility may have been on two different salary grades). As a result, when combining the salary information with our time-and-motion to derive staff costs, we utilized the salary grade most commonly associated with that staff role across all clinics in order to determine which one to use.

**Building Costs**

Building cost data was collected through our facility survey/data collection tool. As such, we collected information on the area of each room and the total facility as well as all of the furniture equipment contained in each room. We calculated the cost per room for each individual room in the facility using a cost of 4200 ZMK/m^2^, as well as the cost of all furniture. In order to calculate an annual cost for the room and furniture, they were depreciated linearly with a 3% discount rate over 30 years and 10 years, respectively. Because the time-and-motion data did not contain refined information with respect to which room each activity was performed in (or the rooms coded did not necessarily align with the room list from our facility survey due to individual rooms having multiple associated “station” references in the time-and-motion data), we averaged the total building cost at each facility and used that as the source with all time-and-motion activities to derive the building cost for each activity. As such, for each activity, time spent performing that activity was multiplied by the per minute cost of the average room in order to determine the building cost. Depending on the distribution of the costs of individual rooms this could produce some under or over-estimates of utilization, but given the data constraints, we felt it was the best way to make this calculation.

**Overhead Costs**

Overhead costs were broken into both administrative and building overhead costs. Administrative costs included the cost of training, community outreach, community drama, security, administrative supplies such as stationery and printing and photocopying, meetings, lunch, transportation, schooling, accommodations, and supervisory costs from the DHO. Building overhead costs included utilities such as phone/airtime, electricity, and water, maintenance for vehicles, plumbing, building maintenance, painting, any maintenance on the surrounding grounds, and cleaning materials. Total annual overhead costs were then multiplied by the %ART number described in the main manuscript in order to allocate which portion of the overhead costs were utilized by the ART clinic.

This % ART of total services value (shown in **Table 1**) was calculated by dividing the total annual ART patient visit volume by the total clinic volume (including non-ART services) plus total number of in-patient bed days plus the total number of surgeries and deliveries performed to estimate what percent of total time of the clinic was dedicated to ART service delivery.

**Waiting Costs**

Waiting times were calculated by subtracting the patient-time of all patient related activities and subtracting that from the total time patients stayed on average at the clinic (departure time minus arrival time). In order to derive a patient waiting cost we multiplied their time waiting by the cost of utilizing 1m^2^ of building space for the total time they were waiting.

**Per Minute Calculations**

Per minute calculations were made by dividing our calculated annual cost figures for staff salaries, building costs, or overhead costs by the total operational minutes for the clinic estimated on the year. The total operational minutes figure was calculated by extrapolating the number of active days at the clinic according to our clinic costing surveys/data collection tool and subtracting the number of observed public holidays. The was then multiplied by the average number of hours the clinics were open for when active (8 hours) and then converted to minutes. For most clinics, the approximate number of operational days was 245 days; therefore, the approximate number of operational minutes was 117600. As such, we divided annual costs by the total number of operational minutes to derive per minute costs for staff, rooms, and overhead.

**Supplies and Laboratory Costs**

Supply costs were extracted from a survey/data collection tool issued to each of the clinics, where clinic research staff estimated the unit cost of clinic supplies for the ART clinics. We asked them to designate how each of these supplies was utilized (for laboratory/diagnostics, ART care, ART Care and other treatment care, other treatment, miscellaneous, etc.), when they were procured, and the total number procured. Additionally, we collected data on the number of laboratory tests run at each of the clinics through the SmartCare database. We received the cost of each relevant laboratory diagnostic test run for the ART patients from CIDRZ research staff. The following laboratory services were included in the cost estimates: CD4 counts, hemoglobin/hematocrit, full blood count, ALT/AST, creatinine, sputum AFB, Chest X-Ray, pregnancy, RPR, TLC, amylase/lipase, and viral load. Because some of the supply procurement schedules featured supplies/chemical reagents featured in the list of laboratory/clinical investigations we did not include their unit costs if the test was featured in the SmartCare database. Instead those costs were simply determined by multiplying the cost of the test as provided by CIDRZ with the total volume of tests run. Additionally, supplies that were not a part of ART treatment or diagnosis or generic (latex gloves, syringes, viral masks, syringes, etc.) were not included in the cost of supplies either.

**Drug Cost Imputation**

In investigating the variation between drug costs, we recognized that one of the clinics (Kalomo) seemed to have some missing data with respect to the prescription regimen. In general, for ART care ARTs should be prescribed at a ratio of 2 NRTIs to every 1 NNRTI. For all other clinics, the most common NNRTI prescribed was Efavirenz. At Kalomo, however, our dispensary records showed that the most commonly prescribed NNRTI at the other clinics, Efavirenz, was not being dispensed at Kalomo, and there was no replacement NNRTI either. In further investigating, we examined the ratio of NRTI to NNRTI across all clinics hoping to find a ratio that centered around 2. **Figure S1** shows the diagnostic chart we used to identify Kalomo as an outlier in terms of its prescription. In order to solve this issue, we then took the average ratio of all other ART medication dispensed to Efavirenz for the other 9 clinics and used that ratio to impute the amount of Efavirenz dispensed at Kalomo. This was done for both the facility-level data and the individual patient-level data. The drug costs reported for Kalomo throughout the manuscript feature the imputed drug costs and not the costs missing Efavirenz.





**Figure S1: ART Dispensary NRTI:NNRTI ratios by clinic**

This chart depicts the ratio or NRTIs:NNRTIs dispensed at each clinic from 2015 until the end of 2017. It is apparent that while most clinics generally maintained a 2:1 ratio over the course of those years, Kalomo was an outlier often fluctuating almost as high as 15:1. This assisted as a diagnostic tool for indicating there was likely a data issue at Kalomo, which we used to justify performing data imputation for the NNRTI Efavirenz.





**Figure S2: Annual Bottom Up Cost per Patient by Cost Category**

This chart depicts the annual bottom up cost for each clinic as broken into each of the cost elements as shown in Table 2. These annual costs were first as a cost per activity, then multiplied by the average number of activities a patient would receive in a year to determine the annual cost for each separate element. All costs are reported in 2016 USD.

**Missing Capacity Costs**

While the ART clinics are technically open 5 days a week, there were usually one or two days in the week when they were not seeing patients or were seeing pediatric patients. As such, the clinics were still running with all staff, but were primarily performing administrative duties or seeing pediatric patients. The missing capacity costs are a way of breaking down these days in which they were seeing very few or no adult patients and allocating them back to per patient costs; however, when going through day-by-day visit totals it is difficult to ascertain what defines a “low capacity” day. As such, we used different threshold criteria in order to calculate missing capacity. Essentially, for each clinic we calculated what the maximum capacity day using the day the clinics saw the most patients in the year as the benchmark. From there we used different thresholds of the percentile distribution of patients seen on days when the clinic was open to determine what the threshold for missing capacity was. Essentially, for the 50% mark, any day in which the clinic was open, but the clinic was seeing fewer than the 50^th^ percentile of clinic patients was considered low capacity.

Next, we determined what the cost per patient of missing capacity was by calculating the cost per day to run the clinic based off building, overhead, and staff costs. This per day cost was divided by the number of patients on the maximum capacity day to derive a missing capacity cost per patient. Then, we totaled the cumulative “amount” of missing capacity by summing up the total number of patients below the missing capacity threshold across the whole year. This total was multiplied by the per patient cost to derive a total missing capacity cost for the year. This total annual missing capacity cost was then divided by the total number of unique patients who visited the clinic for the year in order to allocate across the number of patients who did visit the clinic within the year. We believe these missing capacity costs may highlight inefficiencies or costs of unreported care at the clinic when they are running at full operational capacity, but are not seeing patients, and thus not included in the time-and-motion analysis and thus not the bottom-up costing. Therefore, we included these with the bottom up costs in order to compare with the top-down costs in **Figure 1**. We found that the clinics that often had the largest discrepancies between their bottom up costs and top down costs often had the largest missing capacity costs as well, highlighting that this may be a useful metric for examining clinic inefficiencies. The different missing capacity costs as calculated at different thresholds is featured in **Table S2**. For **Figure 1**, the 95% threshold was used mainly because we believed that on days where the clinics were seeing the maximum number of patients may have been outlier days when the clinic was experiencing an unusually large volume of patients which may have been a greater estimate of “over-capacity” where staff could not adequately handle the number of patients.

**Table S2: Missing Capacity Costs by Missing Capacity Threshold**

| Clinic | 50% | 75% | 90% | 95% | Max |
| --- | --- | --- | --- | --- | --- |
| Clinic 1 | $0.96 | $1.87 | $3.38 | $4.70 | $8.28 |
| Clinic 2 | $0.49 | $0.97 | $1.91 | $2.51 | $5.31 |
| Clinic 3 | $0.76 | $1.49 | $2.89 | $3.62 | $10.14 |
| Clinic 4 | $2.47 | $5.21 | $10.35 | $13.33 | $18.06 |
| Clinic 5 | $0.54 | $1.61 | $3.01 | $4.50 | $17.66 |
| Clinic 6 | $2.13 | $4.73 | $9.44 | $11.89 | $17.08 |
| Clinic 7 | $3.31 | $5.63 | $7.71 | $9.78 | $19.67 |
| Clinic 8 | $1.22 | $3.42 | $5.60 | $8.04 | $19.22 |
| Clinic 9 | $0.80 | $3.53 | $6.83 | $10.52 | $19.85 |
| Clinic 10 | $1.28 | $7.85 | $17.44 | $56.42 | $67.30 |

**Clinic Costs by Geography**

For this analysis we compared the weighted average based on patient populations of the ART clinics for the all of the clinics, the urban clinics, and the rural clinics. As we see, the rural clinics in general have smaller overall bottom up costs, smaller than the urban clinics by $14.81. This difference is largely driven by a $2.09 difference in supply costs, a $3.73 difference in drug costs, and a $12.14 difference in laboratory costs. However, while the rural clinics largely have lower costs in consumables, they feature larger overhead and building costs. This is probably due to having smaller clinic populations that are harder to reach. As such, it may be more difficult for clinics to provide patients with all of the medication, supplies, and laboratory diagnostics they need. Additionally, because the populations are smaller, largely fixed costs in overhead and building costs will be larger on a per patient basis. The rural clinics also have a larger average missing capacity cost, which may indicate that the rural clinics on average are more inefficient in providing services than the urban clinics, likely due to the same reason of having smaller populations and thus more days that are considered missing capacity. This cost breakdown is shown in **Table S3**.

**Table S3: Clinic Costs by Geography**

|  | Overall | Urban | Rural |
| --- | --- | --- | --- |
| Staff | $4.15 | $4.12 | $4.37 |
| Building | $0.62 | $0.41 | $2.00 |
| Overhead | $0.63 | $0.45 | $1.77 |
| Supplies | $6.05 | $6.33 | $4.24 |
| Drugs | $88.24 | $88.73 | $85.00 |
| Laboratory | $17.00 | $18.60 | $6.46 |
| Overall Bottom Up Costs | $116.69 | $118.64 | $103.83 |
| Missing Capacity Costs* | $6.85 | $5.83 | $13.60 |
| Top Down Costs | $130.32 | $131.06 | $125.44 |

**The missing capacity costs described are those taken from the 95% max capacity thresholds*

**Operational Cost Regression Models**

In addition to running univariate regressions on the overall average cost of ART care per patient, we also ran univariate regressions using the operational costs as the dependent variable **(Table S4)**. The operational costs were defined as the average cost per patient of all ART clinic operations not including the ART drug costs and supply costs. We hypothesized that these costs may be better related to the clinic operational characteristics than the ART drug cost (which was also the largest proportion of the average cost of ART care). All of the clinic characteristic variables had a positive relationship with the operational costs, but only two variables were statistically significant (p < 0.05), which were the total number of clinic visits for all ART patients to the clinic and the average number of total annual visits per patient. For each additional increase in 1000 patients, annual operational costs of ART services per patient increased on average by $0.50 (95% CI: $0.02 – $0.98), and for each additional number of total annual visits per patient, annual operational costs of ART services on average increased by $11.99 (95% CI: $0.05 - $23.94)

**Table S4: Univariate Regression Outputs on Dependent Variable: Annual Operational Cost**

| Variable | Change in annual cost of ART services per patient | 95% Confidence Interval | P-value |
| --- | --- | --- | --- |
| Geography (1 = Urban 0 = Rural | $10.52 | (-6.36 - 27.41) | 0.19 |
| Total Number Clinical Visits (per 1000 patients) | $0.50 | (0.02 - 0.98) | 0.04 |
| Unique Patient Volume (per 1000 patients) | $2.14 | (-0.59 - 4.87) | 0.11 |
| Percent of Clinical Services dedicated to ART | $23.62 | (-50.04 - 97.29) | 0.48 |
| Average Number of Total Annual Visits Per Patient | $11.99 | (0.05 - 23.94) | 0.05 |
| Average Number of Annual Clinical Visits Per Patient | $2.70 | (-13.62 - 19.01) | 0.71 |
| Average Number of Annual Pharmacy Visits per Patient | $6.14 | (-1.33 - 13.62) | 0.09 |
| Patients/Counselor Ratio (per 1 additional patient per staff role) | $0.24 | (-0.13 - 0.06) | 0.18 |
| Patients/Clinical Officer Ratio (per 1 additional patient per staff role) | $0.13 | (-0.21 - 0.05) | 0.37 |
| Patients/Nurses Ratio (per 1 additional patient per staff role) | $0.01 | (-0.24 – 0.26) | 0.93 |
| Patients/Pharmacy Technician Ratio (per 1 additional patient per staff role) | $0.23 | (-.17 - 0.64) | 0.22 |
| Patients/Laboratory Technician Ratio (per 1 additional patient per staff role) | $0.05 | (-0.39 - 0.50) | 0.75 |
| Clinical Full Time Employees | $1.12 | (-1.10 - 3.33) | 0.28 |
| Total Clinic Full Time Employees | $1.20 | (-0.22 - 2.61) | 0.09 |
